# Equitable AI: Exploring the role of gender in poverty estimation models using geospatial data

**DOI:** 10.1371/journal.pone.0332193

**Published:** 2025-09-25

**Authors:** Seth Goodman, Katherine Nolan, Rachel Sayers, Ariel BenYishay, Jacob Hall, Mavis Zupork Dome, Edem Selormey

**Affiliations:** 1 AidData, Global Research Institute, William & Mary, Williamsburg, Virginia, United States of America; 2 Ghana Center for Democratic Development, Accra, Ghana; COMSATS University Islamabad - Lahore Campus, PAKISTAN

## Abstract

Household surveys have been the foundation for poverty measurement in developing countries for the past half-century, but the spatial and temporal gaps in these survey data often limit how well anti-poverty programs can be targeted, monitored, or evaluated. To fill in these gaps, analysts and policymakers increasingly turn to machine learning (ML) methods to predict indices of asset wealth from satellite-based and other geospatial data. However, to date, the potential for gender-related differences in these methods’ performance has not been investigated. We implement a frequently used class of ML models (random forests) relying on readily accessible geospatial data and trained on and validated against a widely used source of asset holdings (a recent round of the Demographic & Health Survey in Ghana). By separately aggregating the asset holdings of female- and male-headed households within each survey cluster, we are able to estimate the distinctions in performance of ML models trained on each of these gender-based asset indices. We find that models trained on data from male-headed households achieve an impressive level of predictive accuracy (*R*^2^ = 0.85), while those trained on data from female-headed households achieve reasonable but notably lower accuracy (*R*^2^ = 0.75). Roughly half of this gap appears to be driven in large part by the relatively smaller number of female-headed households in the survey sample. While we cannot rule out that the ML models themselves play a role in creating differences in performance across gender, it appears that these gaps may largely be a reflection of the sampling designs of the underlying survey data used as inputs for these models. Our findings confirm that ML models can be used to extend the spatial and temporal scope of these survey data to populations that were not randomly sampled, even while encouraging larger samples of female-headed households in survey designs to improve the predictive accuracy of ML models for female-headed households.

## Introduction

Poverty measurement is essential to effectively target, monitor, and evaluate policies and interventions aimed at poverty reduction. Traditional approaches to poverty measurement rely on household surveys of a sample of the population. As Deaton [1] documents, efforts to use surveys to record human well-being stretch back to the late 18th century, and have grown in sophistication by integrating statistical theories of random sampling (including clustering and stratification), digital data collection approaches, and other advances.

Today, national statistical offices in many countries rely on household surveys to monitor poverty rates, and a number of international initiatives aim to standardize and support these data efforts. The World Bank’s Living Standards Measurement Survey (LSMS), for instance, has represented the consumption, asset holdings, and other aspects of the material experiences of 6.9 billion people through 115 nationally representative surveys since 1980 [2]. Similarly, since their inception in 1984, the Demographic & Health Surveys (DHS) have been carried out in 90 countries, capturing household well-being, including the types of assets these households own [3]. However, these sample surveys are conducted no more than a few times per decade in most countries and usually include relatively small samples designed to draw inference only for the national population. As a result, these frequency and spatial resolution constraints limit the use of these survey-based poverty estimates for targeting, monitoring, and evaluation.

To fill in these spatial and temporal gaps, remotely sensed geospatial data have been increasingly used over the past two decades. While these efforts initially relied on nighttime lights (NTL) data, more recent work has made use of multispectral daytime imagery to produce estimates of household poverty and other development indicators, such as infrastructure quality and crop yield [4–9]. In addition, remotely sensed geospatial datasets with global coverage of climate [10,11], land cover [12], pollution [13], and other topics are increasingly available to researchers through multiple platforms that readily address data preparation, management, and other technical and computational barriers [14,15]. Beyond remotely sensed data, volunteer geographic information from OpenStreetMap (OSM)—including building footprints, road networks, points of interest, and more— has also been used to produce poverty estimates at fine geographic scales [16,17].

The dramatic expansion of geospatial data in these analyses has naturally pushed researchers to rely on machine learning (ML) methods to maximize the predictive accuracy of these analyses. One subset of ML approaches known as “deep learning” uses satellite images as inputs from which computer vision algorithms (e.g., convolutional neural networks) extract features that correspond with poverty conditions separately measured by household surveys, and then predicts what conditions exist in areas where no surveys were carried out. Deep learning approaches have shown particular promise in their ability to identify relevant features from daytime satellite imagery when used to estimate household assets measured by the DHS [4,16,18–20]. However, deep learning methods can be limited by computational requirements, financial costs, technical knowledge, data accessibility, time to produce, and interpretability of results [17,21–23].

A related strand of literature has explored the use of random forests (RFs) as an alternative, as these have lower computational costs and require less data and preparation [16,17]. RFs are a well-established ML approach for performing classification and regression utilizing ensembles of decision trees [24]. A major distinction between RFs and deep learning approaches is the use of predetermined input features such as NTL by RFs. Tingzon [16] implements both deep learning and RF approaches and shows they produce comparable *R*2 in the Philippines; Htet [17] documents similar results indicating comparability between RF and other ML algorithms in estimating household assets in Myanmar. Both RF and deep learning approaches attempt to estimate asset holdings and other poverty conditions from remotely sensed and other geospatial data in ways that most closely correspond to DHS and other survey-based measures of household assets and well-being.

At the same time, a separate body of work has highlighted major differences in living conditions among men and women within many communities around the world. Male-headed households, on average, have 13% more asset-based wealth and 303% more land-based wealth than female-headed households across a number of countries in Africa, Asia, and South America [25]. In Ghana, the Gender Gap Asset Project has found that women own substantially fewer assets than men, and additionally those assets are generally valued at lower prices [26]. Other efforts to better measure female-specific poverty point to many other ways in which gaps or deprivations can be hidden by household- or community-level aggregates [27,28]. Here, we focus on asset holdings as one key measure of poverty, recognizing that deprivation can occur along many other (potentially correlated) dimensions.

The gender differences in asset holdings in many developing countries naturally lead us to ask whether ML models that predict these asset holdings using geospatial data achieve similar accuracies for women’s and men’s asset holdings and poverty rates. Why might this not be the case? There are multiple points at which gender-related differences could emerge. First, the sample designs of most household surveys such as the DHS are rarely designed to be representative of female-headed households (or to otherwise clearly identify female-controlled assets). Thus, most include only a small number of female-headed households, constraining the ability of ML models to accurately estimate conditions among this group (importantly, this also constrains the precision of traditional estimates based only the survey data as well). Second, most ML models are trained to estimate composite indices of all household assets, and the composition of these indices could disproportionately reflect assets held or used by one gender or another. Third, it is possible that the ML models themselves may be more effective at generating accurate poverty estimates for male-headed households or more specifically, associating poverty with assets more frequently owned by one gender. Taken together, this suggests there is substantial potential for ML-based asset and poverty measures to more accurately reflect conditions in male-headed households than female-headed ones (or, possibly, vice versa).

Our contribution is to provide the first estimates of differences in accuracy rates for ML models between male-headed and female-headed households, as well as to explore the potential sources of these differences. Research and applications of poverty estimates generated using ML have almost exclusively incorporated the full set of DHS households (without gender or other considerations) for training and validating models [16,29]. A recent review of work in this space by Hall [30] found no works that focused on gender, bias, or impacts on potentially vulnerable subpopulation.

**Fig 1:**
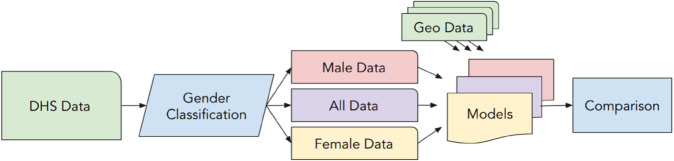
Overview of workflow used to assess influence of gender on ML-based household poverty estimates.

We provide the estimates by utilizing the most widely used and readily accessible sources of geospatial data and household poverty conditions. As noted above, the DHS have been used to generate asset-based estimations of household poverty for decades. We use a recent wave of the DHS conducted in Ghana, a context with notable, documented differences in asset holdings across men and women [26]. To estimate asset holdings, we use RFs, which have been applied in a wide range of applications incorporating remote sensing and geospatial data, including producing poverty estimates [31,32]. Compared to other ML approaches such as XGBoost or Support Vector Machines, RFs are often less prone to overfitting, have fewer and less complex parameters, and are well-suited to parallelization and practical implementation [33]. We train RF models on subsets of the data delineated by the gender of the household head, and then examine how such models perform for the full population (Fig 1).

To foreshadow our results, we find that models trained on data from male-headed households achieve an impressive level of predictive accuracy (*R*^2^ = 0.85), while those trained on data from female-headed households achieve reasonable but notably lower accuracy (*R*^2^ = 0.75). This appears to be driven in large part by the relatively smaller number of female-headed households in the survey sample (they constitute only 1/3 of households). When we randomly drop male-headed households from the sample to balance the household counts across genders, the performance gap between models trained on male- and female-headed household data shrinks by approximately one half. While a variety of modeling choices–such as hyperparameter values, geospatial datasets combinations, and asset index composition–affect the overall model performance, they do not appear to substantially affect the differences in performances across the gender of the household head. Thus, while we cannot rule out that the ML models themselves play a role in creating differences in performance across gender, it appears that these gaps may largely be a reflection of the sampling designs of the underlying survey data used as inputs for these models.

## Country context

Ghana, a West African nation with a population of over 30 million, is a setting in which wealth distribution remains highly uneven. There are significant regional disparities in wealth, with differences between urban and rural communities, as well as among different regions and ethnic groups. Within communities, differences in wealth are influenced by household composition, employment, and education. In particular, avenues for wealth accumulation differ strongly by gender.

In Ghana, wealth is closely linked to the ownership and control of assets, such as land, housing, durable goods, and financial instruments. Asset acquisition occurs primarily through self-purchase and inheritance, with gifts and marriage serving as secondary avenues. These pathways to asset ownership are influenced by both statutory laws and customary practices. Customary inheritance systems, particularly the patrilineal arrangements prevalent in northern and some southern regions, often restrict women’s access to assets, particularly family land and structures. While statutory laws, such as the Intestate Succession Law and the Matrimonial Causes Act, aim to enhance equitable distribution of property by gender upon death and divorce, implementation gaps and social norms often limit their effectiveness. This is especially the case in rural and traditionally governed communities.

Throughout the country, men are more likely to be employed and are more likely to be work in the formal sector [34]. These employment differences translate into income disparities that are reflected in asset ownership. Education levels also differ by gender, with a literacy gap that favors men [34]. Furthermore, women in Ghana are less likely to have financial accounts than men and are less likely to have access to finance and formal credit, further constraining asset accumulation [35]. As such, the avenues for asset accumulation differ by gender, and men and women may strategically invest in different assets.

## Materials and methods

### Data

The units of analysis and the existing asset-based wealth index are from the Demographic and Health Surveys (DHS) round conducted in Ghana in 2014 (the most recent DHS round for Ghana at the time of this research - the 2022 round has since been released). The DHS collects data on population, health, and related information for more than 90 countries and has conducted hundreds of surveys since it began in 1984 (See https://dhsprogram.com for more information). The DHS also collects information on asset measures (i.e., ownership) and calculates the DHS Wealth Index (WI) at the household level [36]. Geospatial information on household location is provided at the cluster level, with each household assigned to a cluster. To preserve anonymity, cluster locations are randomly displaced by up to 2 km in urban areas and up to 5 km in rural areas (1% of rural clusters are displaced up to 10 km). To account for displacement, urban and rural cluster locations are buffered by 2 km and 5 km respectively.

For each household we retain the DHS household identifier, cluster identifier, the DHS WI value, the gender of the head of the household, the associated cluster longitude and latitude, and the buffered unit of observation for the cluster. The head of household gender is based on survey responses to the enumerator conducting the interview for the DHS (i.e., self-reported by the respondent of the household-level survey). The DHS questionnaire used for this survey only provides gender response options of male or female (see https://dhsprogram.com/publications/publication-dhsq6-dhs-questionnaires-and-manuals.cfm).

The DHS WI is calculated using a principle component analysis (PCA) of the various asset measures, allowing for differences in weights for rural and urban households. The assets measured include durable goods, land, and characteristics of the household. The likelihood of owning an asset differs by the gender of the household head for a number of assets. Indeed, in the 2014 wave of the DHS, male-headed households are more likely than female-headed households to have several types of assets, such as land suitable for agriculture (50.9% vs 36.4%), a bank account (51.5% vs 39.3%), and a bicycle (38.7% vs 12.1%). Table 1 reports the difference in ownership rates by gender of household head for the 5 assets with the biggest difference favoring male household heads, as well as for the 5 assets with the biggest difference favoring female household heads.

**Table 1:** This table shows the rates of ownership by the gender of the household head for selected assets. *** indicates a difference is significant at p < 0.01.

Panel A: Top 5 Assets Disproportionately Owned by Male-Headed Households			
**Variable**	**% Ownership in**	**% Ownership in**	**Difference**
	**Male-Headed HHs**	**Female-Headed HHs**	**(Male-Female)**
Bicycle	38.74	12.10	26.64***
Radio	73.15	52.26	20.889***
Land suitable for agriculture	50.85	36.40	14.45***
Motorcycle/scooter	16.71	3.42	13.29***
Video deck/DVD/VCD	39.66	26.52	13.14***
***Panel B: Top 5 Assets Disproportionately Owned by Female-Headed Households***			
**Variable**	**% Ownership in**	**% Ownership in**	**Difference**
	**Male-Headed HHs**	**Female-Headed HHs**	**(Male-Female)**
Charcoal cooking fuel	25.67	39.04	-13.37***
Cement walls	48.99	57.02	-8.03***
Asbestos/slate roofing sheets	59.13	67.13	-8.00***
Ventilated improved pit latrine	31.32	38.80	-7.48***
Public tap/standpipe drinking water	19.01	26.34	-7.33***

Data on NTL and additional geospatial variables in raster formats are aggregated to each buffered DHS cluster using all applicable common aggregation methods (e.g., mean, median, min, max, and sum of pixels in raster data per cluster) [14]. NTL data provides a satellite based measure of illumination on the Earth’s surface, and is available from the Visible Infrared Imaging Radiometer Suite’s (VIIRS) day night band [37] at 500 meter resolution. Other geospatial variables include those related to population count/density, environmental conditions (temperature, precipitation), land cover classification, and pollution (Table 2). Datasets are selected based on broad spatial coverage (to support future work), practical accessibility (publicly available and documented), at least 1 kilometer resolution, and availability of time series data. While quality of the geospatial data - specifically spatial resolution - is relevant in accurately reflecting location specific conditions, the relative scale of the coarsest resolution data (1 km) is well within the bounds of the spatial error introduced by the DHS cluster geolocation displacement and subsequent buffering to ensure the true cluster location is covered (typically 2 or 5 km, as described above).

**Table 2:** Geospatial datasets and years used in random forest regressions.

Data	Year
DHS data and survey cluster locations [43]	2014
Nighttime lights [37]	2013
Precipitation [44]	2013
Temperature [45]	2013
Normalized Difference Vegetation Index [46]	2013
Land cover [12]	2013
Population count [47]	2013
Population density [48]	2015
Travel time to cities [49]	2015
Elevation and slope [50]	NA
Distance to water [51]	NA

The selected datasets reflect a range of human or environmental factors which have been leveraged to gauge socioeconomic conditions in numerous studies of development interventions and resulting impacts [38–42]. Data are extracted from the year prior to the DHS survey (2013) whenever possible to reflect conditions which may have influenced or have a relationship with household asset ownership in the time period leading up to the survey. A total of over 50 features resulted from the datasets and aggregation methods.

The resulting household-level DHS data and associated geospatial features are then used to produce three datasets: 1) a gender agnostic dataset consisting of all household data, 2) a male dataset based on male-headed households, 3) a female dataset based on female-headed households.

### Model design

We utilize the Random Forest Regression (RFR) class from Scikit-Learn (see [16,32] for related implementations) with a grid search to test a range of hyperparameter values (Table 3) and five-fold nested cross validation to validate model performance. In addition to the hyperparameters listed in Table 3, squared error was used as the error criterion across all tests, which utilizes variance reduction as the feature selection criterion for each split in a tree [24]. While RFs are less prone to overfitting when compared to similar ML models, the use of nested cross-fold validation for hyperparameter optimization and testing further reduces the likelihood of overfitting [33]. All models are run across the gender agnostic, male, and female datasets.

**Table 3:** Hyperparameters explored in RFRs.

Hyperparameter	Values	Relevance	Optimal
Number of estimators (trees generated per RF)	300, 500, 1000	More trees increase the complexity of the RF and can improve performance, yet may produce diminishing returns.	500
Max features (considered at a given node for making a split)	"sqrt", 0.33, 0.5, 1.0	Defines what proportion of all features at a given node are considered.	0.33
Max depth (of an individual tree)	7, 10, 15, 20	Greater depth increases complexity and can refine performance, but can lead to overfitting or unnecessarily complicated models.	10
Minimum samples required to split a node	2, 3, 5	If a node has less than this value it is considered terminal. This can help prevent overfitting when dealing with small leaf sizes and/or limited training data.	2
Minimum samples required to produce a new leaf node from a split	1, 2, 4	How many samples must exist in each leaf resulting from a split. Can prevent splits which would result in imbalanced leaves.	1

Two simple feature sets are used initially to establish a baseline. The first uses only NTL, as it is commonly leveraged in linear models to approximate poverty/wealth or economic activity and provides an important reference point. The second uses the raw geospatial location (i.e., the displaced cluster longitude and latitude values), which can reveal generalized spatial trends influencing poverty estimates that may also be reflected in other geospatial variables.

Next, we use all the geospatial features available. Many of these features are likely to be highly correlated with other features in the set. In addition, the models resulting from a large set of features are likely to be more complex and take longer to train, without necessarily resulting in significantly improved performance over more finely tuned models with fewer features. However, by including all features we can develop an initial impression of model capabilities and assess which features are most important to model performance.

Models using the initial three feature sets are trained using the gender agnostic, male, and female data with the full range of hyperparameters in order to gauge initial trends and select optimal hyperparameters (Table 3). The performance of the models is assessed based on the resulting *R*2. While a range of alternative performance metrics exist for ML methods, the use of *R*2 for regression-based models is most frequently used across literature on ML-based poverty estimates. Notably, *R*2 enables comparison with traditional econometric models used for estimating household poverty in related literature.

Based on the results, optimal hyperparameters are selected and features are refined to a more practical set for further analysis. Features which have high collinearity, yet relatively lower feature importance (based on models including all features) are then removed [32].

## Results

We subset the 2014 Ghana DHS data into three sets of household data based on head of household gender and a gender agnostic approach using all household data. The resulting gender agnostic dataset contained 11,835 households across 427 clusters; the male head of household dataset contained 8,008 households across 427 clusters; the female head of household dataset contained 3,827 households across 421 clusters. While around one third of households are classified as female-headed, the effective sample size provided to the RFR model is not significantly impacted since over 98% of survey clusters contain at least one female-headed household.

Much smaller counts of gender-specific households within survey clusters have the potential to create noisier data. This is a result of gender classification taking place at the household level and then being aggregated to the survey cluster level, for which geospatial location information is available. As a robustness check to address variable household counts within clusters, we will rerun the models with the number of households selected for aggregation to the cluster level artificially limited in order to balance gender specific household counts and test the impact of cluster household counts on model performance.

### Model refinement

**Fig 2:**
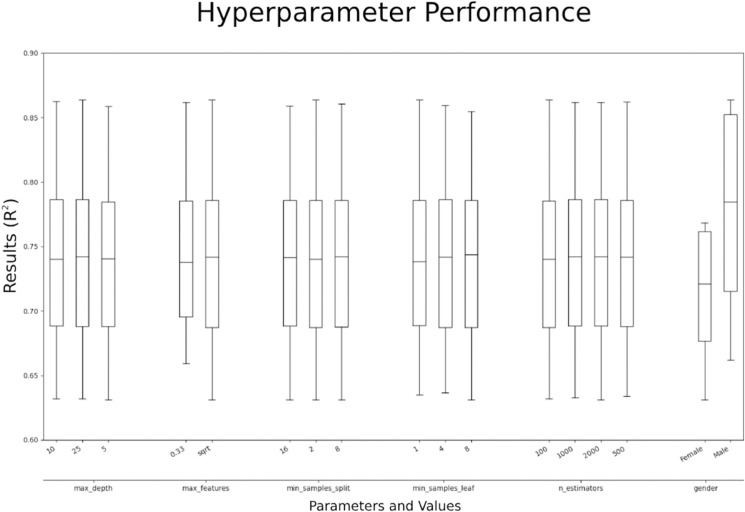
Boxplots of model performance (R2 values) across all hyperparameter tests.

Results from the hyperparameter search revealed that there were relatively minor differences in model performance (based on model *R*2 value) across the range of hyperparameters tested for the gendered and gender agnostic models (Fig 2). A total of 432 hyperparameter variations were tested for each gender/feature combination (results from the full set of tests are available in the GitHub repository linked in the Data Availability Statement). There was, however, a clear shift in the performance of models using male household data compared to models using female household data. Based on a comparison between genders across the range of hyperparameter tests male models were approximately 10% better than female models, as reflected in the difference in median *R*2 value between genders. Male household models produced a median *R*2 value of approximately 0.79 while female household models produced a median *R*2 value of approximately 0.72. Based on the findings from the hyperparameter search, an optimal set of hyperparameter values were selected to be used in subsequent model runs (Table 3).

Three initial feature sets were tested across the male, female and gender agnostic models. The first two models - using NTL or location features alone - serve as a simple baseline for comparing model performance. The third includes the features for NTL and location as well as all other geospatial variables. The results revealed that male household models were very similar in performance to gender agnostic models across all feature sets. Female household model performance was again consistently lower by about 0.1 *R*2. The most efficient feature set (i.e., fewest features for best performance across both gendered models) identified after removing features based on collinearity and feature importance consisted of a subset of geospatial features which includes NTL max and median, average elevation, total precipitation (annual), average normalized difference vegetation index (NDVI - a measure of plant greenness), urban area coverage, forested area coverage, distance to water, and travel time to nearest major city (population over 10k).

Alternative aggregation methods for a single dataset (e.g., mean, median, max, and min of NTL) were a major source of collinearity, along with datasets that can serve as proxies for similar metrics. For example, aggregation approaches for NTL, urban area land cover, population count and density, and distance to major city resulted in many strong correlations as they all typically reflect major population centers. The final feature set sought to minimize heavily correlated features while still retaining a balance of meaningful features that have been established as related to socioeconomic conditions in existing literature.

### Gender

**Fig 3:**
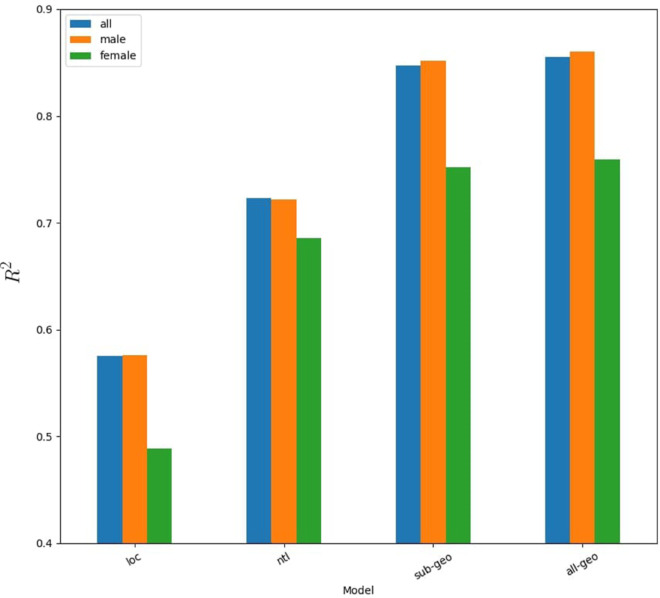
Performance for male, female, and gender agnostic models based on the mean R2 of models trained using features for: cluster locations only (loc), nighttime lights within clusters (ntl), a subset of geospatial variables within clusters (sub-geo), and all geospatial variables within clusters (all-geo).

The result of training and validating gender specific models using the refined set of features, along with the results from the previous tests, revealed that female classified models consistently underperformed (lower *R*2) male classified models, and male models performed similarly to the gender agnostic models (Fig 3). One of the major caveats of the analysis of gender is that the number of households for each gender varies (average within clusters and overall). To evaluate whether imbalance between the household count for genders impacts model performance, we conducted two tests. First, we compare unbalanced and balanced models based on head of household gender. Next, we compare performance when artificially reducing the size of gender agnostic data from all households.

Data for models are balanced at the cluster level prior to cluster aggregation by randomly dropping households within a cluster for the gender with the greater number of households in that cluster. For example, assume using unbalanced datasets cluster A has 30 male households and 20 female households, and cluster B has 22 male households and 26 female households. After balancing, 10 male households would be randomly dropped from cluster A so that there are 20 male and female households. Similarly cluster B would drop 4 female households so that there are 22 male and female households. By balancing at the cluster level we also ensure overall balance between the gender specific samples.

**Fig 4:**
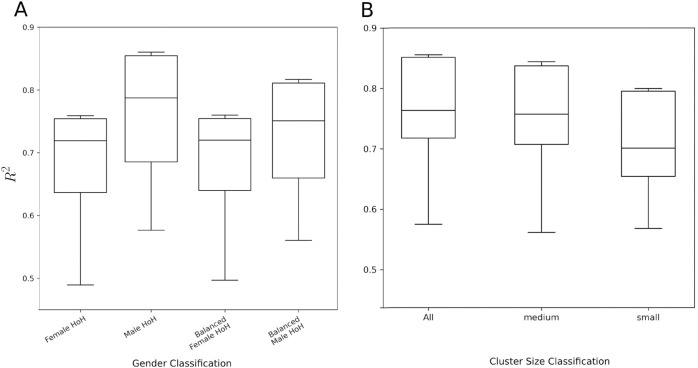
Boxplots of model performance (R2 values) comparing (A) gendered models when using both balanced and unbalanced datasets, and (B) gender agnostic models with household counts per cluster artificially reduced by varying amounts.

Balancing the data for models based on head of household gender showed a visible decrease in performance of the balanced models (Fig 4a). Male models were impacted the most by the balancing (household counts reduced), yet there were some clusters in which there were more female households. As a result, there is a clear decrease from the unbalanced male model to the balanced male model ( 6% average), while the change in the female models was near zero. The changes from the balanced models do reduce some of the discrepancies between male and female model performance, but there is still a gap between the balanced gendered models. Due to the randomization involved in balancing the clusters, the tests were performed five times. Minimal differences were seen, with a range of variation in the *R*2 for the balanced female models of only 0.013 (consistently <1% change relative to unbalanced female model) and 0.042 for the balanced male models (corresponding to a minimum and maximum potential change from the unbalanced male model of 4.2% up to 9.2%).

To more broadly explore how the number of households within a cluster impacts model performance, we artificially reduced the number of households in clusters for the gender agnostic models. The size of the original, unaltered clusters (containing all households, labeled “all” in Fig 4b) varies but averages approximately 28 households. The original clusters were artificially reduced by randomly dropping households at two different levels. The “medium” clusters were created by reducing counts to 19 households, while the “small” clusters were created by reducing to 9 households each.

The performance of the “medium” models showed a relatively small decrease compared to the original models. The “small” model showed a larger decrease in performance relative to the original, but similar relative to the medium (Fig 4b). As a robustness check against the randomization performed, the randomized sampling for the small and medium sized gender agnostic subsets was also performed five times. The results from the robustness test showed a drop in *R*2 for the medium model relative to the original ranging from 1.3% to 3.4%, and a drop in *R*2 for the small model relative to the original ranging from 3.1% to 4.0%.

**Fig 5:**
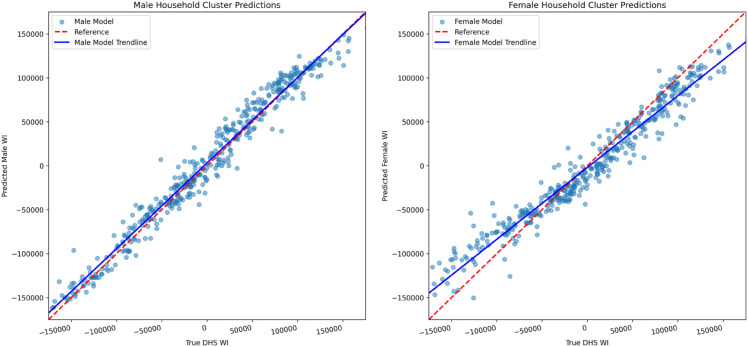
Comparisons of the WI predictions produced for equivalent clusters using gender specific cluster data and models (y-axis) with the original full cluster based DHS WI (x-axis). This provides a visualization of the discrepancy between the baseline existing value used for poverty estimation, and ML-based gender specific poverty estimates. Units based on original WI values produced by the DHS.

The differences in accuracy between the models trained on male- and female-headed households’ data correspond to differences in uncertainty about the true wealth levels of clusters (such differences would be above and beyond any noise due to survey question design and responses). The predictions of cluster WI from models trained on female-headed households are thus likely to be noisier than those from models trained on male-headed households. Fig 5 plots the predicted cluster mean WI of the gender-specific models against the cluster mean WI from the actual DHS derived from all households in the DHS sample. The plots show that predictions for male-headed households (left plot) are much closer to the actual DHS WI trendline than those for female-headed households (right plot). Because we use cluster-level mean WI as our analysis variable rather than household-level WIs, we cannot directly compute household poverty rates from these data. Nonetheless, these results suggest that while poverty rates may not be uniformly biased upwards or downwards based on gender, there is a greater likelihood that women’s poverty rates will be mis-estimated (upwards or downwards) relative to the original DHS WI for the cluster.

### Feature importance

The importance of features provided to the RFR model was also assessed. We used two metrics defining feature importance: mean decrease in impurity (also known as gini importance) indicates a feature’s ability to explain poverty variation across the training samples, while permutation importance reflects how much changes in a feature impact the model’s performance (i.e., decrease the *R*2) [52]. Results from using both methods of measuring feature importance were largely in agreement.

NTL median value and urban area coverage were the two most important features for both models based on male and female households, and population was also consistently a highly ranked (important) feature in all models. While variation in the remaining features were relatively minor, making it difficult to identify clear trends or real-world associations, two distinctions between male and female models are worth highlighting. First, precipitation was more important for male households in terms of rank order (7th for men, 9th for women) but had a relatively small absolute difference (both in terms of decrease in impurity and decrease in *R*2). Similarly, accessibility to major cities was more important when explaining poverty within female household models but the absolute difference was again relatively small. These findings will be explored further in the discussion.

## Discussion

The findings from this research indicate a discrepancy in the effectiveness of ML-based poverty estimates between male and female households. However, there are key limitations in what can be conclusively determined from the current analysis.

**Household gender classification.**

Classifying households by gender is inherently imperfect as the majority of households consist of both men and women who contribute to household assets and wealth. Using the gender of the head of household reflects the most widely used approach in gender-related studies [53] and therefore will be most directly comparable to existing work, but may still obscure specific household dynamics related to key assets used in creating the DHS WI.

For example, a household led by an older male may be practically dependent on younger female family members. Conversely, negative conditions faced individually by a female head of household which impact her asset acquisition (e.g., lower wage than a man in the same occupation) may be obscured by assets purchased by male members of her household. Similarly, information on cultural or regional trends related to who heads a household may be obscured or missing from a simple “head of household” based gender classification.

More granular surveys in which asset ownership and control, as well as subsequent poverty/wealth index metrics, are at the individual level rather than the household level could help improve our ability to train models and evaluate the effectiveness of models at estimating gender-specific poverty. However, the realities of household dynamics will likely mean that it is difficult to isolate individual asset ownership or wealth within a household. As such, improvements to approaches to accurately reflect gender-driven poverty trends within the limitations of the available data will be key to advancing this line of work.

**Gendered sample sizes.**

The results show a relationship between the number of households in clusters and model performance. While tests revealed that this relationship does not account for the full disparity in model performance between male and female households, further work is needed to better understand this relationship. For example, the continued decrease in performance seen when reducing the count of households in the gender agnostic data, may be indicative of a critical threshold in the amount of household data necessary to reasonably reflect the household level characteristics (i.e., poverty) of a given cluster. A cluster with very few households will be more prone to influence of outliers and may be heavily influenced by an individual household rather than the population of that cluster as a whole. When combined with the relatively small cluster sample size available, this has the potential to meaningfully impact model performance.

In addition to testing actual model performance, it may be possible to derive insight from the underlying distributions of the DHS WI within clusters of varying household sizes and the potential impact of “noisier” cluster data based on fewer households. For instance, male household models may perform better in general because their DHS WI values are more closely correlated with full population DHS WI values. Reducing the population size past critical thresholds for female households may no longer reflect a consistent set of conditions for the population that the model is able to adapt to. A potential limitation of the tests we conducted on sample size is the possible variation in distribution resulting from randomly dropping households; future work could conduct many rounds of randomization to explore the robustness of our results. Surveys with more extensive samples, or samples with disaggregated household-level location information, may also be valuable in exploring this further.

**Feature selection and importance.**

Differences in feature importance between male and female models were relatively minor, yet are potentially connected to local conditions. The importance of precipitation in male models may be reflective of men’s differential role in cash crop production and dependence on crop yield for income. The connection between men and agriculture was also seen in the asset ownerships rates from the DHS data, with agricultural land ownership being more common in male-headed households (51%) than female-headed households (36%) as seen in Table 1. This relationship could be explored further by testing if models trained on a subset of male households in areas with greater agricultural activity showed even greater reliance on precipitation.

The importance of access to cities in female models may be because women are less likely to own vehicles or other modes of transportation as reflected in the asset ownership rates from the DHS data. Access to cities could provide access to resources to support their household and/or jobs, both of which would allow them to increase wealth and assets. Exploring female household poverty further when separating between urban and rural locations could reveal additional insights.

More broadly, additional research such as qualitative surveys or other validation exercises to determine if the importance of certain features reflect actual conditions in Ghana would be valuable. Other geospatial features not currently included could also be explored as a way to improve performance or better incorporate specific local conditions into the model. For the features included, we utilized all common aggregation methods that were applicable to the geospatial data, but less common aggregation methods (e.g., skewness, kurtosis) could potentially provide useful information in dataset-specific scenarios.

While the use of explicit features in RFs improves model interpretability relative to deep learning approaches, this does restrict the performance and interpretability of RFs to the variables included. Although the current selection of geospatial features are widely available (spatially and temporally) and capable of facilitating model predictions, they are not necessarily capable of directly exposing country conditions that drive gendered poverty differentials. Approaches to derive the drivers of model performance as they relate to real world conditions are still a relatively nascent and evolving aspect of machine learning research - for poverty estimation [30,54] and more broadly [55] - and reflect a barrier to more fully understanding the role of gender in ML-based poverty estimation approaches.

**Generalizability.**

One of the challenges in training and validating models within a single country using a single DHS round is the limited amount of data. This is particularly notable when working with the geospatial DHS data, as the households are aggregated to clusters. Using only 427 samples (households clusters) to train and validate a model is limiting, and impairs our ability to set aside data for an additional round of out-of-sample testing. Prior work has overcome this issue while simultaneously producing more generalizable models by including data from multiple countries. Typically models are trained and validated on data from all but one country, and then tested on the remaining country’s data [29]. When exploring gender though, this approach may overshadow country-specific gender conditions.

**Alternative wealth indices.**

While the DHS WI is the most commonly used WI in ML-based poverty estimation, alternative WI methodologies exist. The International Wealth Index (IWI) [56] is a compelling option which uses standardized asset weights and fewer, more generalized asset measures. As a result, the IWI can be more useful when comparing poverty/wealth across countries and surveys. Recreating the DHS WI based on gender-specific subsets of the dataset is another option to explore, and may reveal insight into the role of specific assets for estimating poverty metrics for each gender.

**Asset weighting.**

The manner in which assets are weighted to create the WI used in ML-based poverty estimation may affect model accuracy by gender if asset ownership rate differs by the gender of the household head. Due to the differences in Ghana between men and women in employment, education, access to finance, and access to favorable outcomes under statutory and customary law, it is likely that the avenues for asset accumulation differ by gender. This fact, combined with the knowledge that men and women may strategically invest in different assets, makes it likely that the types and values of assets owned by male- and female-headed houses systematically differ.

Since the number of male-headed households outweigh the number of female-headed households, and the asset weights used to generate the WI are derived using a PCA across the entire sample, the resulting WI may be more suitable for measuring the wealth of male-headed households than female-headed households. When regenerating the WI using PCA within the female-headed household subsample, 25.3% of female-headed households were placed in a higher wealth quintile by the DHS WI than by the alternative WI (67.7% remained in the same wealth quintile). This suggests that the sample composition utilized when generating the weights of assets for the DHS WI may lead the WI to systematically overestimate the wealth of female-headed households.

## Conclusion

Household surveys have been the foundation of poverty measurement in developing countries for the past half-century, but targeting, monitoring, and evaluation of anti-poverty programs is often constrained by the spatial and temporal gaps in these survey data. To fill in these gaps, analysts and policymakers increasingly turn to ML methods to predict indices of asset wealth from multispectral imagery and other remotely sensed data, as well as volunteer-based geospatial features. However, these efforts have not accounted for potential gender-related differences in these methods’ predictions. In fact, no known research has explored the relationship between gender and ML-based estimates of poverty, or considered the potential approaches for evaluating the performance of models for subsets of populations traditionally used to train poverty estimation models.

To address this, we implement a frequently used class of ML models (RFs) relying on readily accessible geospatial data and trained on and validated against a widely used source of asset holdings (DHS). By separately aggregating the asset holdings of female- and male-headed households within each survey cluster, we are able to estimate the distinctions in performance of ML models trained on each of these gender-based asset indices. We find that models trained on data from male-headed households achieve an impressive level of predictive accuracy (*R*^2^ = 0.85), while those trained on data from female-headed households achieve reasonable but notably lower accuracy (*R*^2^ = 0.75). Roughly half of this gap appears to be driven in large part by the relatively smaller number of female-headed households in the survey sample. Notably, we do not find that most modeling choices–such as hyperparameter values, geospatial datasets combinations, and asset index composition–affect the gap in performances across the gender of the household head. Thus, while we cannot rule out that the ML models themselves play a role in creating differences in performance across gender, it appears that these gaps may largely be a reflection of the sampling designs of the underlying survey data used as inputs for these models.

The additional measurement error in the models trained on female-headed households translates to greater uncertainty about asset wealth and in resulting estimates of poverty rates. While this does not necessarily bias poverty rates downwards or upwards, it does entail a greater likelihood that poverty rates for female-headed households are more likely to be over- or under-estimated. Again, we note that poverty rates for female-headed households would also be measured with greater uncertainty than male-headed ones even with the survey based measures currently in use.

We draw several key conclusions and implications for policy. First, we find reasonable predictive accuracies even for models trained on female-headed household data alone, suggesting that the ML models do hold substantial promise for efficiently and equitably targeting, monitoring, and evaluating large-scale interventions and policies. Our findings confirm that ML models can be used to extend the spatial and temporal scope of these survey data to populations that were not randomly sampled. At the same time, we find that these models are not able to fully compensate for the smaller samples of female-headed households (or, more generally, for limited observations of female-controlled assets). To increase the predictive accuracy of ML models for female-headed households, the underlying survey data need to include larger samples of these households. This could be achieved by stratification or otherwise over-weighting these households in survey sample designs. In other words, the biases and limitations of the survey data appear to transfer into biases and limitations for the ML models as well. Addressing the ML models’ limitations depends in part on addressing some of those in the survey data used as inputs for these models. In fact, one interpretation of our findings is that women’s poverty is measured more noisily than men’s in both household survey-based and geospatial data-based estimates, and that the accurate targeting, monitoring, and evaluation of poverty for women in particular demands more attention to sample designs that can better power these analyses.

We also recommend that poverty estimates derived from ML or other models routinely report model performance separately by gender. We provide a view on the relative performance of models in one context (Ghana in 2014), but gender differences in model performance likely vary across context. Policymakers relying on these data should request and encourage gender-differentiated model performance estimates that are specific to their policy contexts. Likewise, researchers and analysts producing such data should regularly produce context-specific gender-differentiated performance measures, and explore potential sources for estimated gender gaps.
